# Supplementary material for: Polycistronic Genome Segment Evolution and Gain and Loss of FAST Protein Function during Fusogenic Orthoreovirus Speciation
Source: Viruses. 2020 Jun 29;12(7):702. doi: 10.3390/v12070702 (PMC7412057; doi:10.3390/v12070702)
Supplement: Supplementary file 1 [file viruses-12-00702-s001.zip › Table S1.pdf]

Supplementary Table 1: Accession numbers for orthoreovirus protein sequences used in concatenated structural protein phylograms.

	Core shell	Core RdRp	Core turret	Core NTPase	Outer shell	Core clamp	Outer clamp
**ARV176**	ACH72474	ACH72476	ACH72478	AAT52025	AAW78486	AAC18121	AAC18125
**ARV138**	ACH72473	ACH72475	ACH72477	AAT52024	AAW78485	AAC18122	AAC18126
**ARVtu-A**	ALG03386	ALG03388	ALG03387	ALG03389	ALG03390	ALG03395	ALG03396
**ARVtu-B**	AJW82011	AJW82013	AJW82012	AJW82014	AJW82015	AJW82018	AJW82019
**MdJ18**	AFV52269	AFV52270	AFV52271	AFV52272	AFV52273	AFV52278	AFV52279
**MdZJ2000M**	AGY49078	AGY49079	AGY49080	AGY49081	AGY49082	AGY49084	AGY49085
**Md815-12**	AGO58392	AGO58393	AGO58394	AGO58395	AGO58396	AGO58398	AGO58399
**MdNP03**	AGH25585	AGH25586	AGH25587	AEA92267	AEA92266	AEA92268	ACX37670
**ARVgo-03G**	AFQ62078	AFQ62079	AFQ62080	AFQ62081	AFQ62082	AFQ62087	AFQ62088
**ARVgo-D20/99**	AHL21592	AHL21593	AHL21594	AHL21595	AHL21596	AHL21600	AHL21601
**NBV**	AEQ49382	AEQ49381	AEQ49380	AEQ49383	AEQ49384	AAC18123	AAC18127
**NBVpu**	AEQ49376	AEQ49375	AEQ49374	AEQ49377	AEQ49378	AAR13234	AAR13236
**ARVbu**	AHW40447	AHW40448	AHW40449	AHW40450	AHW40451	AHW40455	AHW40456
**ARVco**	BAQ19493	BAQ19494	BAQ19495	BAQ19496	BAQ19497	BAQ19502	BAQ19503
**MRV1**	AAD42304	AAA47234	AAK57507	CAA42570	AAM10735	AAA47239	CAA43783
**MRV2**	AGG40207	AGG40205	AGG40205	AGG40208	AGG40209	AGG40211	AGG40213
**MRV3**	AJE25913	AJE25911	AJE25911	AJE25914	AJE25915	AJE25918	AJE25920
**RRVbv**	YP009020572	YP009020578	YP009020573	YP009020574	YP009020579	YP009020580	YP009020576
**RRVT**	AOM63684	AOM63686	AOM63685	AOM63688	AOM63689	AOM63692	AOM63693
**BRV**	YP004769549	YP004769548	YP004769547	YP004769550	YP004769551	YP004769553	YP004769554
**MaRV**	YP009246471	YP009246466	YP009246465	YP009246467	YP009246472	YP009246469	YP009246473
**BrRV**	YP003717771	YP003717773	YP003717772	YP003717774	YP003717775	YP003717777	YP003717778
**PRV**	ATE91070	ATE91068	ATE91069	ATE91071	ATE91072	ATE91076	ATE91074

ARV176: Avian orthoreovirus, 176; **ARV138**: Avian orthoreovirus, 138; **ARVtu-A**: Avian orthoreovirus, 19831M09; **ARVtu-B**: Avian orthoreovirus, Reo/PA/Turkey/22342/13; **MdJ18**: Muscovy duck reovirus, J18; **MdZJ2000M**: Muscovy duck reovirus,ZJ2000M; **Md815-12**: Muscovy duck reovirus, 815-12; **MdNP03**: Muscovy duck reovirus NP03/CHN/2009; **ARVgo-03G**: Goose orthoreovirus, 03G; **ARVgo-D20/99**: Goose orthoreovirus, D20/99; **NBV**: Nelson Bay orthoreovirus; **NBVpu**: Pulau reovirus; **ARVco**: Tvarminne avian virus, Corvid; **ARVbu**: Avian orthoreovirus, Pycno-1; **MRV1**: Mammalian orthoreovirus 1, Lang; **MRV2**: Mammalian orthoreovirus 2, TRALAU2004; **MRV3**: Mammalian orthoreovirus 3, T3D-S; **RRVbv**: Reptilian orthoreovirus bush viper, 47/02; **RRVT**: Reptilian orthoreovirus Testudine, CH1197/96; **BRV**: Baboon orthoreovirus; **MaRV**: Mahlapitsi orthoreovirus, 2511; **BrRV**: Broome virus; **PRV**: Piscine orthoreovirus, NOR2012-V3621.
