# Supplementary material for: Polycistronic Genome Segment Evolution and Gain and Loss of FAST Protein Function during Fusogenic Orthoreovirus Speciation
Source: Viruses. 2020 Jun 29;12(7):702. doi: 10.3390/v12070702 (PMC7412057; doi:10.3390/v12070702)
Supplement: Supplementary file 1 [file viruses-12-00702-s001.zip › Table S2.pdf]

Supplementary Table 2: Accession numbers for orthoreovirus outer clamp protein sequences used in phylograms.

Virus Species	Host Species	Isolate/Strain	Accession Number
**ARV**	chicken	176	AAC18125
**MdRVn**	duck	J18	AFV52279
**MdRVc**	duck	815-12	AGO58399
**ARVN**	bulbul	Pycno-1	BAQ19503
**NBV**	bat	NBV	AAC18127
**BRV**	baboon	BRV	YP004769554
**MaRV**	bat fly	2511	YP009246473
**BrRV**	bat	BrRV	YP003717778
**RRV**	Bush viper	RRV-Bv	YP009020576
**RRVT**	tortoise	CH1197/96	AOM63693
