# Supplementary material for: Polycistronic Genome Segment Evolution and Gain and Loss of FAST Protein Function during Fusogenic Orthoreovirus Speciation
Source: Viruses. 2020 Jun 29;12(7):702. doi: 10.3390/v12070702 (PMC7412057; doi:10.3390/v12070702)
Supplement: Supplementary file 1 [file viruses-12-00702-s001.zip › Table S3.pdf]

Supplementary Table 3: Accession numbers for orthoreovirus FAST protein sequences used in phylograms.

Virus Species	Host Species	Isolate/Strain	Accession Number
**ARV**	chicken	176	AAF45151
	chicken	138	AAF45154
	turkey	NC/PEMS/85	ABN46971
	turkey	TX/99	ABN46974
**ARVN**	parrot	PsRVGe01	ABY78878
	bulbul	Pycno1	BAQ19499
**NBV**	bat	NBV	AAF45157
	human	Pulau	YP009507785
**BRV**	baboon	BRV	AAL01373
**MaRV**	bat fly	2511	YP009246474
**BrRV**	bat	BrRV	YP003717780
**RRV**	python	RRV-Py	AAP03134
	bush viper	47/02	YP009020581
**RRVT**	tortoise	CH1197/96	AOM63690
